# Editorial: Genomics and Metabolomics of Microbes in Fermented Food

**DOI:** 10.3389/fmicb.2022.892726

**Published:** 2022-04-19

**Authors:** Madhumita Barooah, Santa Ram Joshi, Bojlul Bahar

**Affiliations:** ^1^Department of Agricultural Biotechnology, Assam Agricultural University, Jorhat, India; ^2^Biotechnology and Bioinformatics Department, North Eastern Hill University, Shillong, India; ^3^School of Sport and Health Sciences, University of Central Lancashire, Preston, United Kingdom

**Keywords:** omics technologies, fermented food, probiotics, amplicon sequencing, LC-MS

Fermented foods and beverages have occupied an integral part of the human diet, with the earliest history dating back to 13,000 BC (Galimberti et al., 2021). During the course of civilization, people have harnessed technical knowhow of food fermentation toward nutritional and health benefits, improved food safety, and organoleptic preferences (Johansen, 2017). Historical evidences suggest that the evolution of fermentation practices was influenced by the properties of the raw materials and prevailing climatic conditions. In addition, the religious, cultural, social, and economic aspects of the local area also had impacts on those practices (Lohani et al., 2007).

The emergence of omics technologies (amplicon sequencing, metagenomics, metatranscriptomics, metaproteomics, and metabolomics) has enabled the present-day researchers in adopting these technologies to redefine the food-based ecosystems. The recent development in omics approaches and affordability of the technologies has led to a paradigm shift in the food science. Use of machine learning and other advanced statistical approaches are extremely helpful in dissecting the fermentation processes and prediction of the metabolic consequences of a food microbial ecosystems (Galimberti et al., 2021). In this edition a collection of omics-based research articles that focus on dissecting the microbial fermentation processes in some of the very important traditional fermented foods and beverages across the world is presented.

One of the world's ancient distilled alcoholic beverages is the Chinese liquor, which is characteristically obtained using *Daqu* fermentation starters. Deng et al. assessed the microbial diversity in *Daqu*—a traditionally prepared starter culture used for the fermentation of a traditional alcoholic beverage called *Nongxiang Baijiu*, and established the correlation between the microbial diversity and flavor compounds. In this study, a temporal metagenomics analysis uncovered the bacterial and fungal diversity associated to volatile production during the fermentation of Daqu (Deng et al.). Li et al. evaluated the fungal community dynamics from six climatically varied Marselan-producing regions in China to establish their role in spontaneous fermentation during red-wine production, and reported the phyla Ascomycota and genus *Aureobasidium* as the dominant fungal group. Based on the high-throughput sequencing data and metabolic profiles, a correlation was established between the potentiality of spontaneous fermentation along with the polyphenol content, and the fungal taxonomic composition in the samples.

Probiotic microorganisms are crucial components of the fermented food products including milk products, fermented vegetables, pickles, and in some non-alcoholic fermented/unfermented beverages. In this context, Gopal et al. employed a metagenomic approach to uncover the presence of probiotic microorganisms in *Kalparasa*—a fresh health drink prepared from the spadix sap of coconut. They reported that fresh *Kalparasa* hosted higher abundance of probiotic *Leuconostoc* followed by equal proportions of probiotic genera *Acetobacter, Gluconobacter*, and *Fructobacillus*. They reported a significant increase in the abundance of *Gluconobacter* with a remarkable decrease in the abundance of *Leuconostoc* after 12 h. This study also suggested that the fermentation of *Kalparasa* was probably driven by symbiotic culture of bacteria and yeasts (SCOBY), particularly acetic acid bacteria and non-*Saccharomyces* yeasts (Gopal et al.). A comparative analysis of traditional and modern fermentation techniques for *Xuecai*—an indigenous fermented vegetable product of China, was described by Zhang et al. Headspace solid-phase micro-extraction followed by gas chromatography-mass spectrometry identified the differential presence of flavor compounds in *Xuecai* produced through traditional and modern fermentation technologies. The group reported a significant correlation between the bacterial community structure (mined through high-throughput amplicon sequencing) and the flavor components for fermented *Xuecai*. Abundances of main flavor compounds such as allyl isothiocyanate, ethyl acetate, 3-butenenitrile, phenol, ethanol, and 3-(2,6,6-trimethyl-1-cyclohexen-1-yl) acrylaldehyde significantly differed between *Xuecai* produced by traditional and modern fermentation (Zhang et al.). Yao et al. described the microbial community structure and metabolites profiles of *Sufu*—a fermented black soybean curd (prepared using *Rhizopus microsporus, Rhizopus oryzae*, and *Actinomucor elegans* as inoculants) at different stages of the fermentation process. The 16S V_3_-V_4_ region and ITS1 region-targeted amplicon sequencing approaches revealed the presence of a core fermenting microbial community comprising *Enterococcus, Enterobacter, Rhizopus*, and *Monascus*. The paper also reports a higher bacterial diversity compared to fungi in *Sufu*. The authors further adopted a network analysis to indicate the positive and negative interactions among bacterial and fungal communities, which are essential to shape the baseline community structure in *Sufu*.

Understanding the microbial interactions in fermentation cultures is an important topic for designing new processes for product development. Xu et al. used integrated transcriptomics and proteomics analyses to describe the protein metabolism in *Lactobacillus helveticus*. *De novo* transcriptome and isobaric tags for relative and absolute quantification proteome analyses were applied to determine how *L. helveticus* utilizes protein. This study described the molecular mechanism of protein utilization, transport and enzymatic hydrolysis in *L. helveticus*, thereby reducing energy consumption. In a similar study, Canon et al. demonstrated the role of specific peptides in the positive interaction between proteolytic and non-proteolytic strains of Lactic acid bacteria (LAB). Tandem mass spectrometry (LC-MS/MS)-based proteomics analysis identified a group of hydrophobic and branched-chain amino acid-containing peptides that were found to be important for the interactions. This study provides crucial information for designing LAB-based starter cultures toward the development of new fermentation products.

## Author Contributions

All authors listed have made a substantial, direct, and intellectual contribution to the work and approved it for publication.

## Conflict of Interest

The authors declare that the research was conducted in the absence of any commercial or financial relationships that could be construed as a potential conflict of interest.

## Publisher's Note

All claims expressed in this article are solely those of the authors and do not necessarily represent those of their affiliated organizations, or those of the publisher, the editors and the reviewers. Any product that may be evaluated in this article, or claim that may be made by its manufacturer, is not guaranteed or endorsed by the publisher.
